# Self-assembled multifunctional neural probes for precise integration of optogenetics and electrophysiology

**DOI:** 10.1038/s41467-021-26168-0

**Published:** 2021-10-07

**Authors:** Liang Zou, Huihui Tian, Shouliang Guan, Jianfei Ding, Lei Gao, Jinfen Wang, Ying Fang

**Affiliations:** 1 CAS Center for Excellence in Nanoscience, National Center for Nanoscience and Technology, Beijing, 100190 China; 2 CAS Center for Excellence in Brain Science and Intelligence Technology, Institute of Neuroscience, Chinese Academy of Sciences, Shanghai, 200031 China; 3 University of Chinese Academy of Sciences, Beijing, 100049 China

**Keywords:** Electrophysiology, Optogenetics, Neuroscience

## Abstract

Optogenetics combined with electrical recording has emerged as a powerful tool for investigating causal relationships between neural circuit activity and function. However, the size of optogenetically manipulated tissue is typically 1-2 orders of magnitude larger than that can be electrically recorded, rendering difficulty for assigning functional roles of recorded neurons. Here we report a viral vector-delivery optrode (VVD-optrode) system for precise integration of optogenetics and electrophysiology in the brain. Our system consists of flexible microelectrode filaments and fiber optics that are simultaneously self-assembled in a nanoliter-scale, viral vector-delivery polymer carrier. The highly localized delivery and neuronal expression of opsin genes at microelectrode-tissue interfaces ensure high spatial congruence between optogenetically manipulated and electrically recorded neuronal populations. We demonstrate that this multifunctional system is capable of optogenetic manipulation and electrical recording of spatially defined neuronal populations for three months, allowing precise and long-term studies of neural circuit functions.

## Abstract (web summary)

The authors present a viral vector-delivery optrode system to integrate optogenetics and electrophysiology. The flexible microelectrode filaments and fiber optics self-assemble in a nanoliter-scale, viral vector-delivery polymer carrier for localized delivery and expression of opsin genes at microelectrode-tissue interfaces.

## Introduction

A central goal in neuroscience is to understand how brain function arises from the activity of neural circuits that consist of various cell types. Addressing this goal requires tools that can selectively manipulate the activity of specific types of cells to assess their roles in neural circuit functions. This has been made possible by recent advances in genetic and optical technologies^1^. In particular, optogenetics allows cell-specific activation or inhibition of neural activity with high temporal precision^2^. Meanwhile, to understand how information is processed in neural circuits, it is essential to record the spatiotemporal patterns of neural activity^3^. Implantable multi-channel microelectrode probes are the most widely used tool to record neural signals at single-neuron, single-spike resolution and have provided profound insights into neural information processing^4–6^. Combining optogenetic stimulation with multi-channel electrical recording can allow for simultaneous cell-type-specific control and spatiotemporal readout of neural activity, thus constituting a powerful tool for the analysis of causal relationships between neural circuit activity and function^7–9^.

The implementation of combined optogenetics/electrophysiology techniques depends on prior delivery and expression of opsin genes in specific types of cells, mostly through stereotaxic injection of viral vectors into a targeted brain region^10^. A second surgery is then required to implant an optical device integrated with microelectrodes (“optrode”) into the same virally transduced brain region for neural stimulation and recording, which, however, causes additional tissue damage and is also susceptible to misregistration error. Moreover, direct injection of viral vectors tends to result in a large and coarse transduction volume due to the rapid diffusion of viral solution in the extracellular space of the brain. As a result, the emitted light from conventional fiber optics typically excites a large neuronal population within a distance of several hundreds of micrometers^10,11^. In behavior studies, the large transduction volumes are valuable for identifying the functional roles of different brain regions^10^. However, the rapid diffusion of the viral solutions can result in dispersed and uneven neuronal expression of opsin genes^12^. This can sometimes result in ambiguous or even contradictory effects in neuronal responses to optogenetic stimulation^7,13^. In combined optogenetics and electrophysiology studies, this deficiency has made functional interpretation of recorded neuronal activity rather tenuous^9,11^. In addition, stable neural modulation/recording over long time courses, i.e., weeks to months, is of central importance in understanding many brain functions, including learning and memory^14^. Hence, there is a need to develop strategies for high-precision, long-term integration of optogenetics and electrophysiology in the brain.

In this study, we describe a multifunctional system for long-term optogenetic stimulation and multi-channel electrical recording of spatially defined neuronal populations in the brain. Each system consists of an array of flexible microelectrode filaments (MEFs) and fiber optics, both of which are embedded in a nanoliter-scale, viral vector-delivery poly(ethylene glycol) (PEG) carrier through an efficient self-assembly process driven by elastocapillary interactions. After in vivo implantation in the mouse brain, the dissolution of PEG carrier allows highly localized viral delivery and neuronal expression of opsin genes within a distance of ~100 µm from flexible microelectrodes, thus ensuring high spatial congruence between optogenetically manipulated and recorded neuronal populations. We demonstrate that our multifunctional probes enable optogenetic inhibition of electrically recorded neuronal populations with substantially improved precision compared to conventional methods (91% versus 24%). Owing to their one-step surgery and low invasiveness, chronically implanted probes further allow simultaneous optogenetic stimulation (inhibition/activation) and multi-channel recording of spatially defined neuronal populations for three months.

## Results

### Design and self-assembly of multifunctional VVD-optrodes

**Fig. 1:**
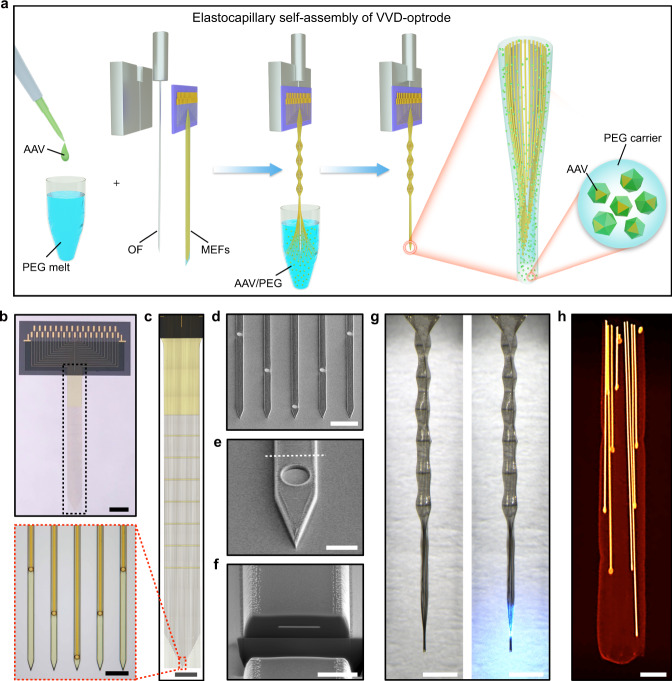
Self-assembled multifunctional VVD-optrodes. **a** Schematics of the elastocapillary self-assembly process of a VVD-optrode. **b** A MEF array. Scale bar, 2 mm. **c** Zoom-in views of the plane-mesh-filamentous structure in the black dashed box in (**b**). Scale bar, 1 mm and 50 μm (magnified view). **d** SEM image (tilted at 45°) of the microelectrode filaments of a MEF array. Scale bar, 50 μm. **e** Enlarged SEM image (tilted at 45°) of a microelectrode filament. Scale bar, 10 μm. **f** Focused ion beam-polished cross-section along the white dashed line in (**e**). Scale bar, 5 μm. **g** A self-assembled VVD-optrode without (left) and with blue light illumination (right), respectively. Scale bar, 1 mm. **h** False-color micro-CT image of a self-assembled probe. Scale bar, 50 μm.

The viral vector-delivery (VVD)-optrodes, consisting of flexible MEFs, fiber optics, and viral vectors, were constructed via an elastocapillary self-assembly process^15^ (Fig. 1a). The MEFs were fabricated, as previously described^16^, using microfabrication techniques (Supplementary Fig. 1). Each MEF array consisted of 33-channel 100-nm thick gold microelectrodes that were distributed along 3-μm-thick, 12-μm-wide, and 4.5-mm-long polyimide (PI) filaments (Fig. 1b–f). The MEFs had a bending stiffness of only $7.8\times {10}^{-14}\,{{{{{\rm{N}}}}}}\cdot {{{{{{\rm{m}}}}}}}^{2}$, approximately five orders of magnitude lower than that of state-of-the-art silicon probes^6^. The flexibility and high-aspect ratio of the MEFs can greatly facilitate their spontaneous self-assembly under elastocapillary forces. Moreover, the plane-mesh-filamentous structure design of the MEF array allows a gradual change in bending stiffness, thus resisting fracture failure during deformation. Microelectrodes on the MEF array were electrically addressable through substrate-supported bonding pads, and the electrical connection between the array and measurement electronics was made *en masse* using flip chip-bonded flexible printed circuits (FPCs).

To construct a VVD-optrode (Fig. 1a and Supplementary Fig. 1), an adeno-associated virus (AAV) vector solution was added in a molten PEG (MW 4000) bath at a volume ratio of 1:9, with the temperature kept at 45 °C. An optrode, consisting of a MEF array aligned with an optical fiber (OF), was immersed in the molten AAV/PEG mixture and progressively withdrawn into the ambient air. During withdrawal, the flexible MEFs were pulled together by the capillary force of the molten PEG and spontaneously self-assembled on the surface of the OF^15–17^. Meanwhile, the molten PEG that was captured within the enclosed flexible filaments quickly solidified in ambient air, resulting in the formation of a thin and stiff PEG fiber with embedded optrode and AAV vectors. A representative VVD-optrode consisting of a sharpened OF and 33 microelectrode sites as illustrated in Fig. 1g. The base diameter of the sharpened OF was approximately 200 μm, and its depth of illumination was approximately 1 mm. The front-ends of the flexible MEF were positioned to extend *ca*. 500 μm beyond the tip of the OF. As a result, the self-assembled probe was gradually tapered from a diameter of *ca*. 220 μm to *ca*. 90 μm. X-ray micro-computed tomography (micro-CT) imaging further revealed the 3D packing of the flexible filaments and longitudinally distributed microelectrodes embedded in a nanoliter-scale PEG carrier (Fig. 1h).

Because of its biocompatibility and biodegradability, PEG has been extensively used in implantable biomedical devices and as carriers for drug delivery (Supplementary Figs. 2 and 3). In our study, the solidified PEG in a self-assembled VVD-optrode acted as both mechanical support required for optrode penetration into the brain and also a nanoliter-scale carrier for spatially precise viral vector delivery in the mouse brain. After implantation, PEG became gradually dissolved and degraded in the extracellular space of the brain (Supplementary Fig. 4), allowing the release of viral vectors for localized transduction of nearby neurons, the OF for the delivery of light, and the MEFs for multi-channel extracellular recording. In addition, the small sizes and high flexibility of our MEFs greatly reduced mechanical mismatch and micromotion-induced inflammation responses at microelectrode-tissue interfaces^18–22^. Immunohistochemical staining results confirmed that chronically implanted MEFs elicited minimal neuronal cell loss in mouse brains at 5 weeks after implantation (Supplementary Fig. 5). The close proximity of neurons to the microelectrodes on MEFs is essential for reliable recording of neuronal activity because the amplitude of extracellular action potential (AP) signals decays exponentially with the distance from neurons^23^.

### Highly localized transgene delivery and expression in mouse brain

**Fig. 2:**
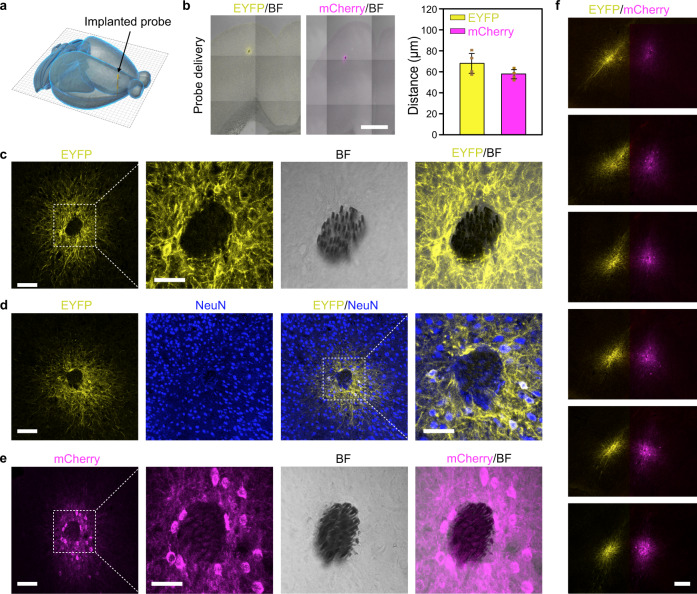
Spatially confined transgene delivery and neuronal expression in mouse brain. **a** A schematic showing an implanted AAV-delivery probe in mouse brain. **b** Transduction characteristics of AAV9 vectors delivered through self-assembled probes. Images (left) are overlays of EYFP/mCherry fluorescence and bright field. Data (right) represent the mean ± SD (*n* = 4 mice in each group). Scale bar, 1 mm. **c** Spatially localized EYFP expression around implanted MEFs. The MEFs can be seen in the bright field image. **d** Neuronal expression of EYFP around MEFs. **e** Spatially localized mCherry expression of an implanted AAV9-hSyn::ChR2-mCherry-delivery probe at 3 weeks. Scale bars in (**c**-**e**), 100 and 50 μm (magnified views). **f** Spatially confined transgene expression of two closely implanted self-assembled probes in the same mouse brain. One probe was loaded with AAV9-hSyn::eNpHR3.0-EYFP (left), and the other was loaded with AAV9-hSyn::ChR2-mCherry (right). Scale bar, 200 μm.

Spatially precise transgene delivery and expression in the mammalian brain is of central importance for both basic and translational neuroscience. We have examined the in vivo transduction characteristics of viral vectors delivered by our self-assembled probes implanted in mouse brains. We chose AAV serotype 9 (AAV9) because it is widely used for transgene delivery in the mammalian brain and is currently examined in clinical trials for the treatments of neurological disorders^24^. Two types of AAV9 vectors were used here, one carried the gene encoding halorhodopsin version 3.0 (eNpHR3.0), an inhibitory opsin, fused to an enhanced yellow fluorescent protein (EYFP) under the control of neuron-specific human synapsin-1 (hSyn) promoter (AAV9-hSyn::eNpHR3.0-EYFP), and the other carried the gene encoding channelrhodopsin-2 (ChR2), an excitatory opsin, fused to mCherry under the control of hSyn promoter (AAV9-hSyn::ChR2-mCherry). We implanted self-assembled probes, consisting of MEFs and AAV vectors, into the secondary motor (M2) and ventral orbital (VO) cortex of wild-type mice (Fig. 2a). Three weeks after implantation, the mouse brains were sectioned horizontally, i.e., perpendicular to the implanted probes, and analyzed for eNpHR3.0-EYFP or ChR2-mCherry expression. As illustrated by a representative 30-μm-thick brain slice with an implanted AAV9-hSyn::eNpHR3.0-EYFP-delivery probe in Fig. 2b, c, eNpHR3.0-EYFP expressing cells were exclusively confined to the vicinity of implanted MEFs. Immunohistochemical labeling with neuronal marker NeuN further confirmed that the eNpHR3.0-EYFP-expressing cells were neurons (Fig. 2d and Supplementary Fig. 6). Spatially confined neuronal expression of ChR2-mCherry was also obtained using implanted AAV9-hSyn::ChR2-mCherry-delivery probes (Fig. 2e), as well as AAV9-CaMKIIα::ChR2-mCherry-delivery probes (Supplementary Fig. 7), demonstrating the general applicability of our system for highly localized transgene delivery and neuronal expression in the brain. Moreover, to illustrate the versatility of our system for spatially precise transgene expression, we implanted two self-assembled probes, one containing AAV vectors that express yellow fluorescent eNpHR3.0-EYFP protein and the other containing AAV vectors that express red fluorescent ChR2-mCherry protein, into the M2/VO cortex of the same mouse brain, at a distance of only ~500 μm. We found highly localized and non-overlapping transduction of neurons around the two closely spaced MEF arrays along their implantation tracts (Fig. 2f).

In our approach, the total volume of viral vectors in an implanted probe was approximately 10 nL. The small volume of viral vectors contained in the self-assembled probes, together with their encapsulation by solid-state PEG polymer carrier, greatly reduced their spreading distance in the extracellular space of the brain and allowed for efficient opsin expression in neurons around the microelectrodes^25^. The average distance of virally transduced neurons from our implanted probes was only 68 ± 9 μm for eNpHR3.0-EYFP and 58 ± 4 μm for ChR2-mCherry (Fig. 2b), respectively. This unique feature ensures high spatial congruence between optogenetically stimulated and electrically recorded neuronal populations in our system.

### Simultaneous optogenetic stimulation and electrical recording of neuronal activity

**Fig. 3:**
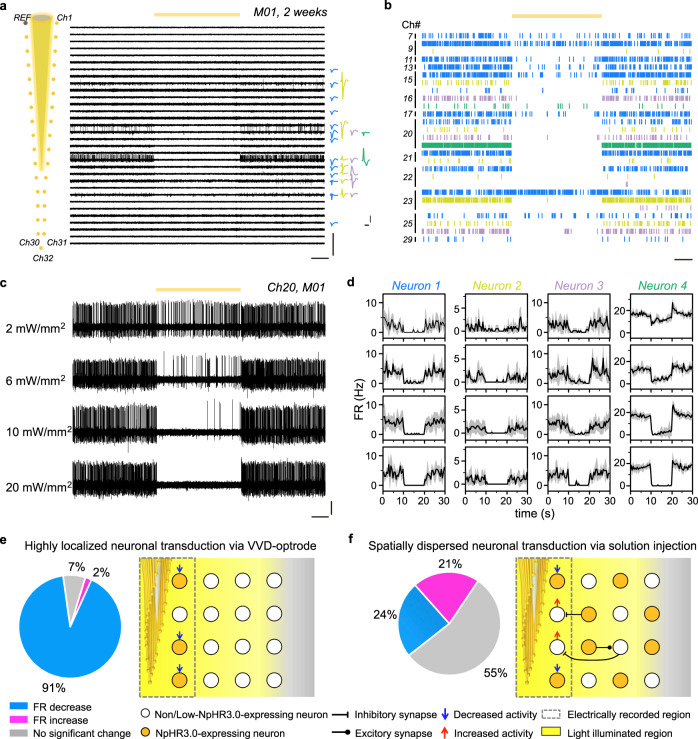
Electrical recording during optogenetic inhibition of eNpHR3.0-expressing neurons. **a** AP traces were recorded in the M2/VO cortex of mouse M01 by an AAV9-hSyn::eNpHR3.0-delivery optrode at 2 weeks after implantation (left), from which 27 neurons were isolated (right). The yellow line indicates the duration of 10-s yellow light stimulation. Scale bar, 800 μV (vertical, left), 2 s (horizontal, left), 100 μV (vertical, right), and 1 ms (horizontal, right). **b** Spike rasters of 27 neurons in M01 during yellow light stimulation. Scale bar, 2 s. **c** AP traces were recorded by an example channel (Ch20) in response to 10-s yellow light stimulation at 2–20 mW/mm^2^. Scale bar, 100 μV (vertical) and 2 s (horizontal). **d** Averaged firing rates of neurons recorded by Ch20 under 2–20 mW/mm^2^ stimulation (from up to down). The firing rates were binned at 2 Hz. **e**, **f** Percentage of neurons with a decrease or an increase in firing rates during 20-mW/mm^2^ stimulation (*n* = 2 mice in each group). Neurons were transduced with eNpHR3.0 via VVD-optrodes in (**e**) and solution injections in (**f**), respectively. The right panels are schematics showing the neuronal transduction characteristics of AAV-delivery probes and conventional solution injections.

We have tested the capability of our self-assembled multifunctional probes to integrate optical stimulation and multi-channel electrical recording of neuronal activity in mouse brains. For optogenetic inhibition of neuronal activity, VVD-optrodes carrying AAV9-hSyn::eNpHR3.0-EYFP vectors were implanted in the M2/VO cortex of wild-type mice. After 2 or 3 weeks of eNpHR3.0-EYFP expression, the MEF arrays were routed to 36-pin Omnetics connectors via custom printed circuit boards (PCBs) for signal readout, and the sharpened OF was connected to a laser for optical stimulation. Continuous yellow light (589 nm, 10-s duration) was delivered through the OF to inhibit eNpHR3.0-expressing neurons. We obtained high-quality multi-channel recording with the flexible MEF arrays in awake, head-fixed mice during optogenetic stimulation. For example, a 33-channel MEF array implanted in a mouse (M01) isolated the activity of 27 putative individual neurons (Fig. 3a). Light illumination at 20 mW/mm^2^ led to rapid and reversible suppression of spiking activity in most recorded neurons (*p* < 0.05, two-tailed unpaired *t* test) (Fig. 3b). We found that the suppression efficiency of neuronal activity depended on the laser power density, as illustrated by the recording of a representative microelectrode channel (Ch20) in Fig. 3c. Four neurons were isolated from the microelectrode channel, and their firing rates all decreased with an increased laser power density (from 2 to 20 mW/mm^2^) (Fig. 3d). At a laser power density of 2 mW/mm^2^, the firing rates of the 4 neurons were decreased by 96, 77, 86, and 31%, respectively. When the laser power density was increased to 20 mW/mm^2^, all 4 neurons were strongly inhibited (>95%) by light illumination.

Overall, we were able to isolate 43 neurons from 2 mice (M01 and M02) during a recording session (Supplementary Fig. 8). We found that a great majority of the recorded neurons (91%) showed light-induced activity suppression at 20 mW/mm^2^, and only a few neurons showed no detectable change in spiking (7%, 3 neurons) or increased spiking (2%, 1 neuron) upon light illumination (Fig. 3e). The three neurons showing no light response may be due to low eNpHR3.0 expression level, and one neuron showing light-induced activation could result from reduced inhibition by nearby presynaptic eNpHR3.0-expressing interneurons.

For comparison, we carried out combined optogenetics/electrophysiology in mice in which eNpHR3.0 was expressed in neurons via conventional stereotactic injections of AAV solutions^7,13^. Each experiment required two surgeries. Firstly, a 300 nL AAV9-hSyn::eNpHR3.0-EYFP solution was stereotaxically injected into the M2/VO cortex of a mouse brain using a syringe. This resulted in a large transduction distance of 589 ± 255 μm from the injection site. At 2 weeks after the first surgery, an assembled optrode consisting of a 33-channel MEF array and a tapered OF was implanted into the same virally transduced brain region. Simultaneous optogenetic stimulation and electrophysiological recording were performed 1 week after the second surgery. Notably, yellow light illumination (20 mW/mm^2^) induced complex activity responses in the recorded neurons (*n* = 33), with only 24% of the neurons showing light-induced activity suppression, and a majority of recorded neurons showed no detectable change in spiking (55%) or increased spiking (21%) upon light illumination (Fig. 3f). Similar results have also been reported in previous studies using optrodes based on microwire bundles^7^ or silicon probes^13^ and can be attributed to the spatially dispersed and uneven neuronal expression of opsin genes by conventional injection methods (Supplementary Fig. 9). Misalignments between microelectrodes and viral solution injection sites can result in low opsin expression in neurons at microelectrode-tissue interfaces, which can lead to low optogenetic modulation efficiency of electrically recorded neurons. In addition, strong coupling between neurons can result in ambiguous or even contradictory effects in neuronal responses to optogenetic silencing^9,11^. For example, silencing of inhibitory interneurons with high opsin transfection can result in increased spiking of excitatory cells with low or no opsin transfection through synaptic effects (Fig. 3f). Distinct from previous studies, our VVD-optrodes allowed highly localized delivery and neuronal expression of opsin genes at microelectrode-tissue interfaces. This can enable efficient optogenetic modulation of electrically recorded neurons, a feature highly desirable for dissecting neural circuits.

### Long-term optogenetic stimulation and electrical recording of neuronal activity

**Fig. 4:**
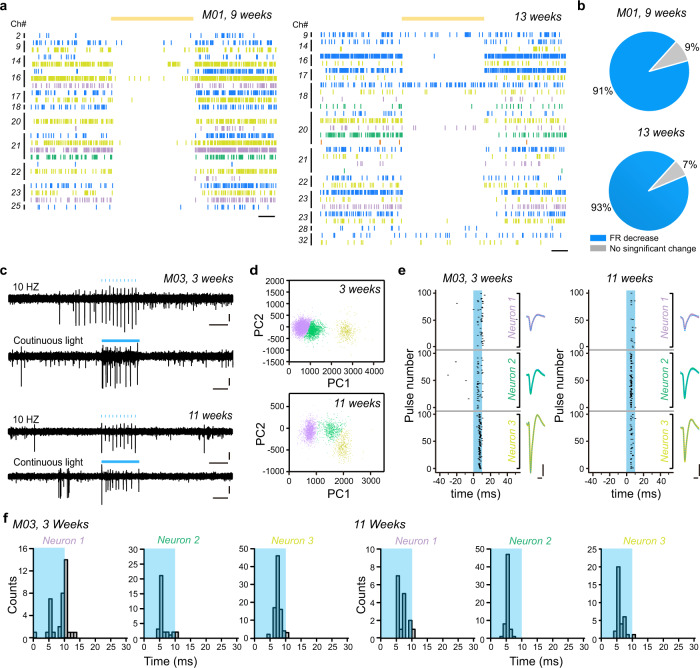
Long-term optogenetic stimulation and electrical recording. **a** Spike rasters of neurons recorded by an AAV9-hSyn::eNpHR3.0-delivery optrode implanted in mouse M01 during 10-s, 2-mW/mm^2^ yellow light stimulation at 9 (left) and 13 weeks (right) after implantation, respectively. Scale bar, 2 s. **b** Percentage of neurons with different responses in firing rates during 2-mW/mm^2^ yellow light stimulation at 9 (up, 25 neurons) and 13 weeks (down, 30 neurons) after implantation, respectively. **c** Example AP traces in response to 10 Hz, 10-ms blue light pulses, and 0.5-s continuous illumination at 3 and 11 weeks after implantation. The traces were recorded by a microelectrode channel (Ch24) of an AAV9-hSyn::ChR2-mCherry-delivery optrode implanted in the M2/VO cortex of mouse M03. Light stimuli are indicated by blue bars, and the power density was 20 mW/mm^2^. Scale bar, 50 μV (vertical). **d** Principal component analysis (PCA) of sorted waveforms. **e** Raster plots showing the spiking times of recorded neurons relative to the onset of each 10-ms light pulse during 10 Hz stimulation at 3 and 11 weeks after implantation, respectively. Scale bar, 0.5 ms (horizontal) and 50 μV (vertical). **f** Latency between the light onset and the first spike at 3 and 11 weeks after implantation. Blue boxes indicate the 10-ms laser pulses.

We have tested the performance of our multi-functional VVD-optrodes for long-term optogenetic stimulation (inhibition/activation) and electrical recording in mouse brains. Multi-channel recording results during optogenetic inhibition by the same MEF array implanted in mouse M01 at 9 and 13 weeks are shown in Fig. 4a and Supplementary Fig. 10. Notably, high-quality AP signals were obtained with the same MEF array without any position adjustment at both time points, from which 25 and 30 neurons were isolated at 9 and 13 weeks, respectively. In addition, a great majority of the recorded neurons (84% at 9 weeks and 87% at 13 weeks, respectively) showed light-suppressed activity at a low laser power density of 2 mW/mm^2^ (Fig. 4b), presumably due to enhanced opsin expression in the mouse brain over the extended periods of time^10,26^.

For long-term optogenetic activation and electrical recording of neuronal activity, we implanted self-assembled VVD-optrodes carrying AAV9-hSyn::ChR2-mCherry into the M2/VO cortex of wild-type mice (Supplementary Fig. 11). Multi-channel local field potential (LFP) and AP signals during blue light illumination were recorded by implanted MEF arrays (Supplementary Fig. 12), from which 44 neurons were isolated from 2 mice (M03 and M04). Representative light-evoked signals recorded by a microelectrode channel (Ch24) of the MEF array implanted in mouse M03 at 3 weeks and 11 weeks after implantation are shown in Fig. 4c, d. The good SNR of the spike waveforms confirmed that the microelectrode remained close to the recorded neurons over extended periods of time. In addition, blue light pulses rapidly activated the spiking of ChR2-expressing neurons at both time points (Fig. 4e, f and Supplementary Fig. 13). Collectively, these results highlight the capability of our VVD-optrodes for long-term optical stimulation (inhibition/activation) and electrical recording of neuronal activity in the brain.

## Discussion

In this study, we have developed a multifunctional system for high-precision integration of optogenetic manipulation and electrophysiological recording of neuronal activity in the brain. Our probes were fabricated through an efficient and scalable elastocapillary self-assembly method that allowed simultaneous encapsulation of flexible MEFs, fiber optics, and AAV vectors within nanoliter-scale, tissue-dissolvable PEG carriers. The small volume of AAV vectors, together with their encapsulation within the solid PEG carrier, permits highly localized transgene expression at microelectrode-tissue interfaces in the brain. Moreover, implanted MEF arrays elicited minimal neuronal cell loss because of their small sizes and high flexibility. As a result, our system enabled simultaneous optogenetic stimulation and electrical recording from spatially defined neuronal populations over extended periods of time. This capability is especially desirable to study the functional organization and integrative mechanisms of the cortical columns in sensory areas, such as the barrel cortex.

The highly localized transgene expression of our multifunctional system makes it a promising tool for studying neural circuit functions. Further expansion of their capability could be achieved by integrating the MEF arrays with on-chip amplifying and multiplexing circuitry^6^. This can reduce the number of interconnection leads and allow simultaneously recording from large populations of neurons. In addition, recent studies have demonstrated that microscale, light-emitting diode (μLED) devices^8,27^ could provide precise optogenetic stimulation with minimal invasiveness. Incorporating these optoelectronic devices into our system could further enable multi-site optogenetic stimulation of neuronal activity. Notably, a wide variety of biomolecules and therapeutic agents could be embedded into the nanoliter-scale PEG carrier of our system (Supplementary Fig. 14), enabling spatially confined drug/gene delivery and modulation of cellular functions at microelectrode-tissue interfaces^28^. Our method can also be applied to other flexible electrodes, including carbon fiber (CF) microelectrodes^19,29^ that are commercially available to most labs (Supplementary Fig. 15). These developments will pave the way toward a versatile platform for high-precision neural circuit analysis and engineering in basic and translational neuroscience.

## Methods

### Fabrication of MEF arrays

The MEF arrays were fabricated using standard microfabrication processes, as shown in Supplementary Fig. 1. The main fabrication steps are as follows: (1) A 100-nm-thick aluminum sacrificial layer was patterned on a silicon wafer (Silicon Valley Microelectronics Inc., Santa Clara, CA) with photolithography (MA6 Mask Aligner, SUSS MicroTec Group, Garching, Germany) and magnetron sputtering (Lab 18, Kurt J. Lesker Co. Ltd., Frankfurt am Main, Germany). (2) A 1.5-μm-thick layer of PI (U-Varnish S, UBE Industries, Ltd., Tokyo, Japan) was spin-coated as the bottom insulating layer and cured at 200 °C for 2 h in a vacuum. (3) The wafer was spin-coated with LOR 3A lift-off resist (MicroChem Corp., Westborough, MA) and baked at 170 °C for 10 min. Shipley S1813 positive photoresist (Microposit, the Dow Chemical Company, Midland, MI) was then spin-coated and baked at 115 °C for 3 min. The LOR 3A/S1813 layers were patterned with photolithography and developed in MF CD-26 (Microposit, the Dow Chemical Company) for 30 s. The wafer was then treated with reactive ion etching (Etchlab 200, SENTECH Instruments GmbH, Berlin, Germany) for complete removal of any resist residue (etching conditions: 20 sccm O_2_, 200 W, 20 Pa). (4) A 5-nm-thick layer of chromium and a 100-nm-thick layer of gold was deposited subsequently with e-beam evaporation (Ohmiker-50B, Cello Technology Co. Ltd., Hsinchu, Taiwan) to form the microelectrodes, interconnects, and bonding pads. (5) A second 1.5-μm-thick PI layer was spin-coated onto the wafer and cured at 200 °C for 2 h to obtain the top insulating layer. (6) AZ4620 positive photoresist (Hoechst Celanese Corp., Irving, TX) was spin-coated on the wafer and patterned by photolithography. The exposed PI was then etched with RIE (etching conditions: 20 sccm O_2_, 200 W, 20 Pa, *ca*. 7 min) to form the filament structure and also expose the recording sites and bonding pads. (7) The bonding pads of the 33-channel devices were flip-chip bonded to custom 0.2-mm-thick FPCs (33 positions, 0.3-mm pitch, Shenzhen D&X Electronic Technology Co. Ltd., Shenzhen, China) using an anisotropic conductive film (MF331, Hitachi Ltd., Tokyo, Japan). (8) The aluminum layer was etched with 1 M FeCl_3_ solution to release the flexible MEFs from the substrate. (9) Electrodeposition of nano-platinum on the recording sites of the MEFs was performed in an aqueous solution of 12 mM chloroplatinic acid (Shanghai Macklin Biochemical Co., Ltd., Shanghai, China). A constant voltage of −0.1 V was applied for 20 s by a Gamry Reference 3000 electrochemical workstation (Gamry Instruments, Warminster, PA). The impedance of the MEFs was reduced to 50–100 kΩ at 1 kHz after nano-platinum electrodeposition. (10) The devices were washed with deionized water 3 times, sterilized with 70% ethanol, and then transferred to deionized water.

### Device structure characterizations

Optical images of as-fabricated devices and self-assembled probes in Fig. 1b, c, g were obtained using an Olympus LEXT OLS4000 laser scanning confocal microscope (Olympus Corporation, Olympus LEXT OLS4000 version 2.2.3). Scanning electron microscopy (SEM) images of the devices in Fig. 1d–f were collected using a Nova Nanolab 200 FIB/SEM dual beam system (FEI Corp., Hillsboro, OR) at 5 kV, and the cross-sections of the filaments were prepared by focused ion beam (FIB) milling. Micro-CT images of the probe in Fig. 1h were acquired using Zeiss Xradia 520 versa (Carl Zeiss X-ray Microscopy Inc., Oberkochen, Germany) with an 80 kV laboratory X-ray source.

### Self-assembly of VVD-optrodes

The AAV vectors, including AAV9-hSyn::eNpHR3.0-EYFP and AAV9-hSyn::ChR2(H134R)-mCherry were purchased from Hanbio Co. Ltd. (Shanghai, China). We firstly carried out differential scanning calorimetry (DSC) analysis (MDSC Q2000, TA Instruments Inc.) to determine the melting characteristics of our PEG 4000 samples (95904-250G-F, Sigma-Aldrich Co. LLC, St. Louis, MO) and 10% H_2_O/90% PEG 4000 mixtures. As shown in Supplementary Fig. 1, the PEG 4000 polymer melts over a fairly broad temperature range from 50 to 60 °C, and a mixture of 90% PEG 4000 with 10% H_2_O has a lower melting range of 20–45 °C. The self-assembly experiments were thus carried out as follows^30^: (1) The MEF arrays were washed with deionized water 3 times, sterilized with 70% ethanol, and then transferred to deionized water. (2) To construct an optrode, an MEF array and a sharpened OF (Plexon Inc., Dallas, TX) were aligned together with a homemade 3D printed holder. The holder was secured to a micromanipulator (RWD Life Science Co. Ltd., Shenzhen, China), and the device was transferred into a 100 °C PEG 4000 bath. The device was withdrawn from the molten PEG bath into air at a speed of ~0.5 mm/s. The flexible MEF filaments spontaneously assembled on the OF surface under the capillary force of the molten PEG, forming an optrode. The front ends of the MEFs were located ~500 μm below the tip of the OF. (3) The AAV vectors were adjusted to desired titers with phosphate-buffered saline (1× phosphate-buffered saline (PBS), PH = 7.4, HyClone Laboratories, Logan, UT), and then added into 50 °C molten PEG with a volume ratio of AAV and PEG to be 1:9. The temperature of the AAV/PEG mixture was then reduced to 45 °C. Note that AAV vectors are stable below a temperature of 65 °C. (4) The front end of the optrode was dissolved in deionized water and re-assembled in a 45 °C molten AAV/PEG bath to form a VVD-optrode. The VVD-optrodes can be stored at 4 °C for up to 3 days without any observable effect on the transduction efficiency. The volume of the implanted PEG carrier was calculated by the diameter and implanted length of the probe. The diameter of our probes was about 90–120 μm, and the implantation depth was 1.7 mm. So the total volume of the implanted PEG carrier was 8–17 nL. The total weight of our system, including an assembled probe, a 3D-printed holder, and a fiber optic ferrule, was 1.5 ± 0.2 g.

### In vivo implantation of multifunctional probes in mouse brains

All animal procedures were approved by the Animal Care and Use Committee of the National Center for Nanoscience and Technology, China. Male C57BL/6 mice (Vital River Laboratory Animal Technology Co. Ltd., Beijing, China) were used. The mice were 7–8 weeks old and weighed 20–30 g at the time of viral injection and probe implantation. The mice were individually housed in a temperature and humidity controlled environment under a 12 h:12 h light:dark cycle, with ad libitum access to food and water.

For intracortical implantation of the self-assembled probes, mice were anesthetized with intraperitoneally administered pentobarbital sodium (0.01 g/mL; MYM Technologies Ltd., Hyderabad, India). An anesthetized mouse was placed in a standard rodent stereotaxic apparatus frame (RWD Life Science Co. Ltd., Shenzhen, China) and fixed with two ear bars. Chlortetracycline hydrochloride eye ointment (Shuangji Pharmaceutical Co. Ltd., Beijing, China) was applied to both eyes to prevent eye drying. A heating pad was placed under the mouse to maintain its body temperature at 36–37 °C, and the anesthesia depth was monitored by testing the paw pinch reflex before the start of surgery. A custom-made stainless steel plate was fixed on the mouse skull with tissue glue (3 M China Ltd., Shanghai, China). A stainless steel wire was secured onto the steel plate and implanted into the contralateral hemisphere of the brain as the ground electrode. A square hole of *ca*. 1 mm^2^ was drilled into the skull above the target brain region, and the dura was carefully removed. A self-assembled probe was fixed on a micromanipulator, and then stereotaxically implanted into the targeted brain region at a speed of *ca*. 1 mm/s. The hole on the skull was filled with silicon elastomer Kwik-Sil (World Precision Instruments, Sarasota, FL) before the probe was fixed onto the skull with dental acrylic (Shanghai New Century Dental Materials, Shanghai, China). After surgery, the mice were allowed to recover on a 37 °C heating pad for 1 h before returning to their home cages. Intraperitoneal injection of antibiotic drug was given to the mice for 3 days after the surgery. The implantation depth of our self-assembled probes is currently limited by the dissolution rate of the PEG to be ~5 mm. To target deeper brain regions in larger animals, a mixture of PEG with poly(lactic-co-glycolic acid) (PLGA) or poly(lactic-co-glycolic acid)-Polyethylene glycol (PLGA-PEG) copolymers can be used. This can allow us to target brain structures up to 1 cm.

To examine AAV transduction characteristics, self-assembled probes consisting of MEF arrays and AAV vectors were implanted into the M2/VO cortex of mice. Specifically, a mouse was first anesthetized, and a square hole of *ca*. 1 mm^2^ was drilled into the skull above the left and right M2/VO cortexes. The dura was removed carefully. A self-assembled probe containing AAV9-hSyn::eNpHR3.0-EYFP or AAV9-hSyn::ChR2(H134R)-mCherry was fixed on a micromanipulator, and then stereotaxically implanted into the M2/VO cortex of the mouse brain according to the following stereotaxic coordinates: anteroposterior, +2.50 mm; mediolateral, +0.80 mm; dorsoventral, −1.70 mm. To examine the transduction characteristics of conventional stereotactic injection, 300 nL AAV solutions at a titer of 10^12^ v g/mL were injected into the M2/VO cortex of mice (*n* = 2). The AAV solution was injected with a 10 µL microinjector (Hamilton, Reno, NV; 1701RN; cat. no. 7653-01) assembled with a G34 syringe needle (Hamilton, Reno, NV; 34 gauges, cat. no. 207434). The injection rate was 0.1 μL min^−1^ and was controlled by a PUMP 11 ELITE Nanomite (Harvard apparatus, Inc., Holliston, MA). After injection, the syringe was left in place for 10 min before retracting the needle to avoid solution backflow.

### Tissue imaging and immunohistochemistry

All confocal fluorescence images were acquired on a Zeiss LSM710 confocal microscope (Carl Zeiss X-ray Microscopy Inc., Oberkochen, Germany, with data collection software Carl Zeiss ZEN 2010B SP1 version 6.0.0.485) and analyzed by Carl Zeiss ZEN 2.3 SP1 (black) version 14.0.0.201 and ImageJ 1.47v. (1) Fluorescence images of EYFP and mCherry were taken 3 weeks after AAV vector delivery and transduction. The mice were first anesthetized with an intraperitoneal injection of 1% pentobarbital sodium (wt/vol) and transcardially perfused with 1× PBS and 4% paraformaldehyde (wt/vol), successively. After that, the mice were decapitated, and the brains were carefully removed. The brains were incubated in 4% paraformaldehyde overnight for fixation and then dehydrated in 30% sucrose (wt/vol) in PBS overnight. The brains were then embedded in OTC and frozen at −20 °C for 2 h before being sectioned into 30-μm-thick slices perpendicular to the implanted probes (Leica Biosystem, Austria). The slices were mounted on glass slides, and fluoromount G (Southern Biotechnology Associates, Inc., Birmingham, AL) was used to protect the fluorescent proteins from quenching. 514-nm and 543-nm lasers were used as the excitation sources for EYFP and mCherry, respectively. (2) For immunostaining analysis, brain slices were incubated in 0.3% Triton X-100 at 25 °C for 15 min after frozen sectioning to increase the permeability of the membrane. The slices were blocked with 3% bull serum albumin (BSA) for 1 h at room temperature to occupy the nonspecific binding sites. Then the slices were transferred into primary antibodies (1:200 dilution, MAB377 for NeuN, Millipore; 1:1000 dilution, HPA056030 for GFAP, Sigma; 1:200 dilution, GTX127939 for CAMKIIa, GeneTex; 1:1000 dilution, 226003 for c-Fos, SYSY) overnight or 24 h (c-Fos) at 4 °C. After being rinsed 5 times with 1X PBS, the slices were incubated in fluorophore-conjugated antibodies for 2 h. Goat anti-mouse immunoglobulin G (IgG) [heavy and light chains (H + L)] secondary antibody 405 (1:1000 dilution, A-31553, Thermo Scientific) and goat anti-mouse IgG (H + L) secondary antibody 633 (1:1000 dilution, A-21050, Thermo Scientific) were used for neurons staining. Goat anti-rabbit IgG (H + L) secondary antibody 488 (1:1000 dilution, A-11008, Thermo Scientific) was used for GFAP and c-Fos staining. Goat anti-rabbit IgG (H + L) secondary antibody 405 (1:500 dilution, A-31556, Thermo Scientific) was used for CAMKIIa staining. The slices were incubated in 1 μg/mL 4′,6-diamidino-2-phenylindole (DAPI, 1:1000, C1002, Beyotime) for the staining of nuclei. After being rinsed with 1× PBS 5 times, the slices were mounted on glass slides. Fluoromonut G was used to protect the fluorophores from quenching. Confocal fluorescent images were acquired using 405/488/633-nm laser as the excitation source. The average transduction distance of each mouse in Fig. 2b was firstly calculated using five brain slices, which were then averaged across all mice to obtain the mean and standard deviation of the transduction distance.

### Simultaneous optogenetic stimulation and electrical recording

(1) For simultaneous optogenetic stimulation and electrical recording experiments, self-assembled VVD-optrodes were implanted into the M2/VO cortex (position according to bregma: anteroposterior, +2.50 mm; mediolateral, +0.80 mm; dorsoventral, −1.70 mm) of male C57BL/6 mice (*n* = 4). Two mice were implanted with probes containing AAV9-hSyn::eNpHR3.0-EYFP vectors for optogenetic inhibition, and the other two mice were implanted with probes containing AAV9-hSyn::ChR2-mCherry vectors for optogenetic activation. All stimulation and recording experiments were performed on awake mice on a one-weekly or two-weekly basis. (2) For comparison, two mice were transduced by AAV9-hSyn::eNpHR3.0-EYFP vectors via conventional stereotactic injection in the M2/VO cortex. Two weeks after viral solution injection, self-assembled optrodes consisting of MEF arrays and OFs were implanted into the same brain region. Stimulation and recording experiments were performed on awake mice at 1 week after optrode implantation.

Light illumination was generated by a diode laser (Hangzhou Newdoon Technology Co., Ltd.) and delivered to the mouse brains through the OFs of the implanted probes (Doric Lenses, Quebec, Canada). (1) Continuous yellow light (Aurora-220-589) was used for the optical inhibition of AAV9-hSyn::eNpHR3.0-EYFP expressing mice. The light was delivered in a square wave pattern of variable power density from 2 to 20 mW/mm^2^. For each stimulation and recording session, each mouse was subjected to four consecutive stimulation trials. Each stimulation trial consisted of a 10-s stimulation epoch followed by a 20-s light-off epoch. (2) For mice expressing AAV9-hSyn::ChR2-mCherry, blue light (Aurora-220-473) was delivered in either pulsed or continuous mode. For pulsed stimulation, 10-ms blue light pulses were delivered at 10 Hz. Pulsed stimulation at each frequency consisted of 10 consecutive trials, each of which had a 1-s pulse train with 9-s inter-train intervals. For continuous stimulation, 0.5-s light-on epochs were separated by 9.5-s light-off epochs, and the stimulation was repeated 10 times during a recording session. The light power density was 2–20 mW/mm^2^.

Electrophysiological data were collected by a 128-Channel Data Acquisition System (Blackrock Microsystems, LLC, Salt Lake City, UT, Central application version 7.0.16.0). The MEF arrays of the implanted probes were connected to a 32-channel Headstage (Blackrock Microsystems, LLC, Salt Lake City, UT), and a microelectrode channel of the array was used as the reference electrode (REF). The device yield in our study was 86 ± 5% at 1 week after implantation. The failure of some of the microelectrodes might be due to broken FPC interconnections. The signals from our MEF arrays were multiplexed through the digitizing amplifier. Electrophysiological signals were digitized at a sampling frequency of 30 kHz. The raw recording data were filtered in the 250–5000 Hz frequency range to extract AP data, and a high-cut 250 Hz filter to extract LFP data by Neuroexplorer (NeuroExplorer data analysis application version 5.1.1.8). Motion/chewing artifacts were removed from the recording data by using the common average referencing method. Spike sorting of the filtered AP traces was performed by amplitude discrimination using Offline Sorter software (Plexon, Dallas, TX, Offline Sorter application version 4.4.0). The spike amplitude threshold was set as 4–5*σ*, where *σ* was the standard deviation. All sorted spikes were clustered by principal component analysis. Clusters with an L-ratio of less than 0.2 and an isolation distance greater than 15 were included as single units. The average isolation distance and L-ratio of 27 isolated clusters in Fig. 3a are 66.70 ± 57.70 and 0.06 ± 0.07, respectively. The average isolation distance and L-ratio of 55 isolated clusters in Fig. 4a are 32.45 ± 17.89 and 0.17 ± 0.37, respectively. The spiking times of all spikes assigned to each cluster were then used to compute the interspike interval (ISI) histogram under a bin size of 1 ms. Any cluster that showed refractory period violations (>0.1% of spikes with ISI < 1 ms) was considered as noise and excluded from further analysis.

For firing rate analysis of the eNpHR3.0-expressing neurons, and two-tailed unpaired *t* tests (^*^P = 0.05) were used to determine whether significant differences existed between the baseline and the stimulation firing rates. The stimulation firing rate was defined as the average firing rate during 10-s yellow light stimulation epochs (*n* = 4). The baseline firing rate was calculated from a 100-s pre-stimulation period. The pre-stimulation period was divided into 10 of 10-s epochs, and the baseline firing rate was defined as the averaged firing rate during each 10-s baseline epoch. For spiking time analysis of the ChR2-expression neurons. Raster plots were constructed for each neuron by collecting the spikes occurring in a total of 100 ms before and after stimulation by Matlab R2009b.

### Statistics and reproducibility

Statistical significance in all experiments was evaluated using Microsoft Excel. The significance of differences between the means in experimental groups and control groups was analyzed using the two-tailed unpaired *t* test. The level of significance was set to *p* < 0.05. The experiments in Fig. 1d–f were repeated five or more times with similar results. The experiment in Fig. 1h was repeated three times with similar results. The experiments in Fig. 2c–f were repeated from four animals with similar results. The experiments in Supplementary Figs. 2a, b; 3a, b; 4b, c; 5a; 6; 7; 9a, b; 11; 12f; 14a, b; 15b–d were repeated three or more times with similar results.

### Reporting summary

Further information on research design is available in the Nature Research Reporting Summary linked to this article.

## Supplementary information

Supplementary information

Reporting Summary

## Data Availability

The electrophysiology data generated in this study have been deposited on Figshare: 10.6084/m9.figshare.c.5055200.v1. Data used to plot Fig. 2b; Fig. 3d–f; Fig. 4b, f; Supplementary Fig. 5b; Supplementary Fig. 6; Supplementary Fig. 13 in this paper have been provided in the Source Data file. Additional raw data generated in this study are available from the corresponding author upon reasonable request. Source data are provided with this paper.

## Source data

Source Data
