# Supplementary material for: Self-assembled multifunctional neural probes for precise integration of optogenetics and electrophysiology
Source: Nat Commun. 2021 Oct 7;12:5871. doi: 10.1038/s41467-021-26168-0 (PMC8497603; doi:10.1038/s41467-021-26168-0)
Supplement: Supplementary file 1 — Supplementary information [file 41467_2021_26168_MOESM1_ESM.pdf]

SUPPLEMENTARY INFORMATION for

Self-assembled multifunctional neural probes for precise integration of optogenetics and electrophysiology

Liang Zou[1,2,3]†, Huihui Tian[1]†, Shouliang Guan[1,2,3], Jianfei Ding[1], Lei Gao[1,2,3], Jinfen Wang[1], Ying Fang[1,2,3]*

[1]CAS Key Laboratory for Biomedical Effects of Nanomaterials and Nanosafety, CAS Center for Excellence in Nanoscience, National Center for Nanoscience and Technology, Beijing 100190, China.

[2]CAS Center for Excellence in Brain Science and Intelligence Technology, Institute of Neuroscience, Shanghai Institutes for Biological Sciences, Chinese Academy of Sciences, Shanghai 200031, China.

[3]University of Chinese Academy of Sciences, Beijing 100049, China.

†These authors contributed equally to this work.

*Corresponding author. E-mail: fangy@nanoctr.cn

This PDF file includes:

Supplementary Fig. 1. MEF array fabrication and elastocapillary self-assembly of VVD-optrode.

Supplementary Fig. 2. Biocompatibility of PEG.

Supplementary Fig. 3. TEM characterizations of AAV virus in PEG.

Supplementary Fig. 4. *Ex vivo* implantation and dissolution of a nanoparticle-loaded MEF/PEG probe.

Supplementary Fig. 5. Chronic tissue responses to MEFs at 5 weeks after implantation.

Supplementary Fig. 6. Neuronal transduction of self-assembled probes.

Supplementary Fig. 7. Opsin expression in excitatory neurons at microelectrode-tissue interfaces.

Supplementary Fig. 8. Optogenetic inhibition and electrical recording in mouse M02 at 3 weeks after implantation.

Supplementary Fig. 9. Transduction comparison between self-assembled probes and solution injections.

Supplementary Fig. 10. Long-term optogenetic inhibition and electrical recording in mouse M01.

Supplementary Fig. 11. Localized c-Fos activation at microelectrode-tissue interfaces.

Supplementary Fig. 12. Simultaneous optogenetic activation and electrical recording by implanted AAV9-hSyn::ChR2-mCherry-delivery probes in mouse brains.

Supplementary Fig. 13. Latency between the light onset and the first spike of ChR2-expressing neurons at 3 weeks post implantation.

Supplementary Fig. 14. A brain slice with an implanted nanoparticle-delivery probe.

Supplementary Fig. 15. VVD-optrode based on carbon fiber (CF) microelectrodes.

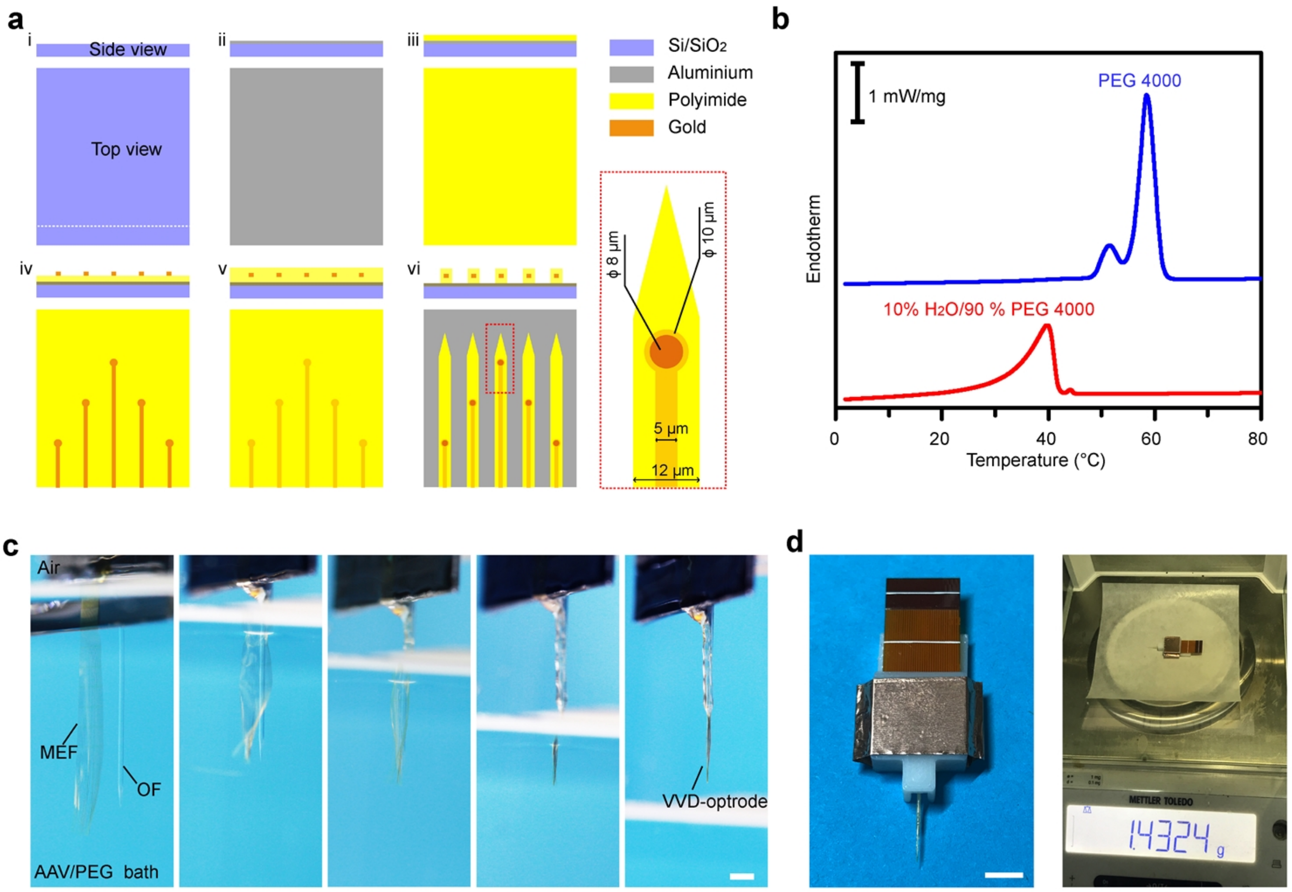

Supplementary Fig. 1. MEF array fabrication and elastocapillary self-assembly of VVD-optrode. **a**, Side and top views of the key fabrication steps: (i) The side view of a silicon wafer substrate corresponding to the cross section indicated by the white dashed line in the tope view; (ii) Photolithography (PL) patterning and deposition of aluminum sacrificial layer; (iii) Spin-coating and curing of bottom 1.5-µm-thick polyimide (PI) layer; (iv) PL patterning and deposition of gold microelectrodes, interconnects, and bonding pads; (v) Spin-coating and curing of top 1.5-µm-thick PI layer; (vi) PL patterning and RIE to define PI filaments and expose recording sites at the front end and bonding pads at the rear end. **b**, Differential scanning calorimetry (DSC) analysis of PEG 4000 polymer and 10% H_2O/90% PEG 4000 mixture. The DSC curves were measured when the samples were heated to 90 °C at a rate of 5 K·min^{-1}. Curves were shifted vertically for clarity. **c**, Photographs of the elastocapillary self-assembly process of a 33-channel VVD-optrode. Scale bar, 1 mm. **d**, A self-assembled VVD-optrode with a 3D-printed holder and a fiber optic ferrule. The average weight of our system is 1.5 ± 0.2 g. Scale bar, 0.5 cm.

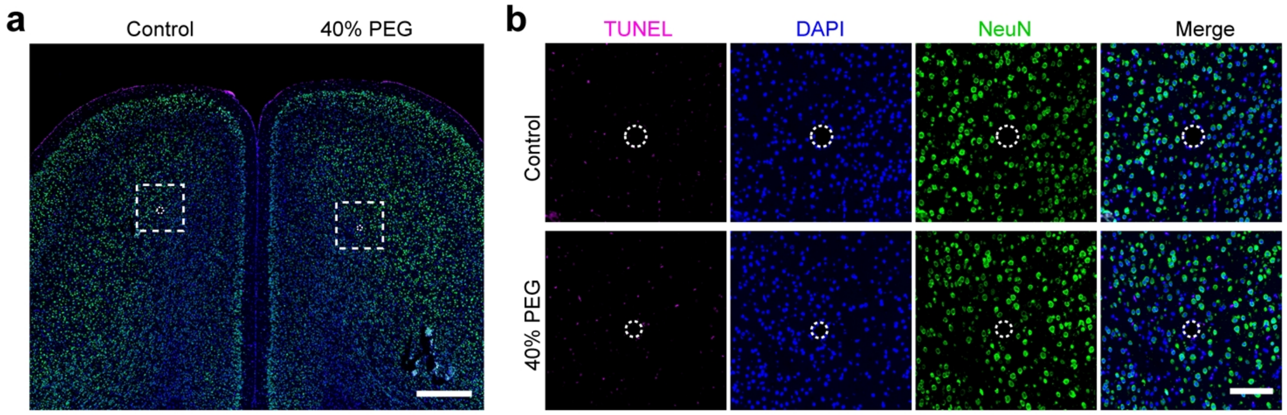

Supplementary Fig. 2. Biocompatibility of PEG. a, Immunohistochemical staining of a brain slice that was injected with 300 nL PBS (left side) and 40% PEG (right side) solutions, respectively. The 4-μm-thick brain slice was prepared at 3 weeks after injections and labeled for nuclei [4', 6-diamidino-2-phenylindole (DAPI), blue] and neurons (NeuN, green). Cell apoptosis was assessed by terminal deoxynucleotidyl transferase dUTP nick end labeling (TUNEL, magenta). The dashed circles highlight the injection sites. Scale bar, 500 μm. **b**, Enlarged views in the white dashed boxes in **a**. Scale bar, 100 μm.

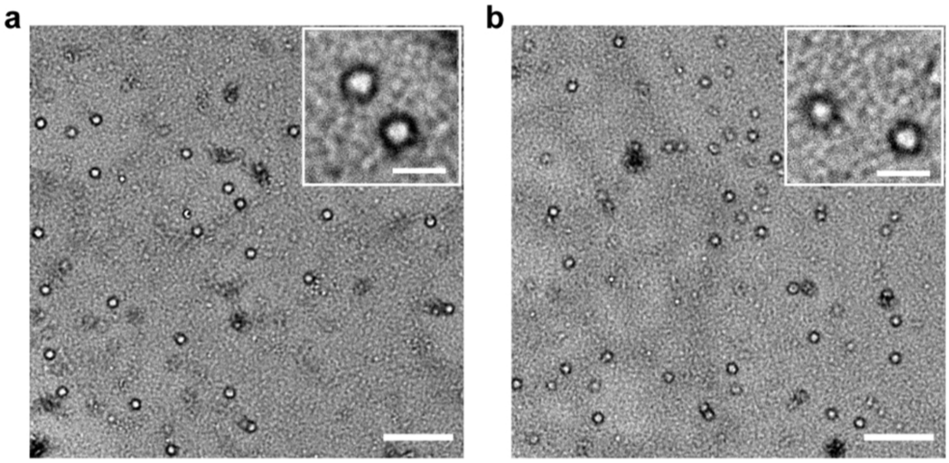

Supplementary Fig. 3. TEM characterizations of AAV virus in PEG. Negative stain of AAV9-CMV::GFP virus from untreated AAV9-CMV::GFP (**a**) and AAV9-CMV::GFP/PEG solutions (**b**), respectively. Insets are enlarged images of the AAV virus particles. TEM samples of AAV vectors were prepared as following: copper grids (250-mesh, coated with a formvar-thin carbon film) were loaded with 10 μL pure AAV solution or AAV/PEG solution at a titer of 10^{11} v·g/mL, and then stained with 5% uranyl acetate. The samples were viewed with an Ht-7700 (Hitachi, Ltd) transmission electron microscope. Scale bars, 200 nm and 50 nm (inset).

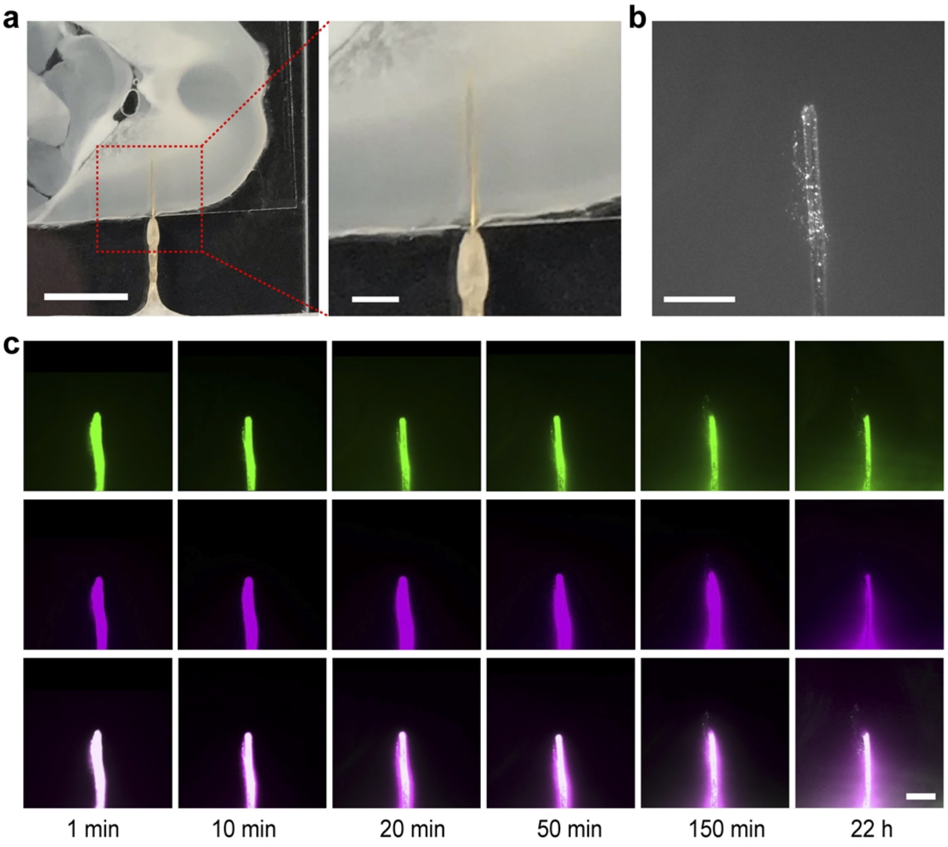

Supplementary Fig. 4. ***Ex vivo*** **implantation and dissolution of a nanoparticle-loaded MEF/PEG probe.** **a**, Photos of an assembled MEF/CdSe-ZnS/RhB-PEG probe implanted in a 500 µm-thick mouse brain slice. The PEG 4000 molecules in the assembled probe were covalently conjugated with Rhodamine B (RhB), and 12-nm-diameter CdSe-ZnS core-shell nanoparticles with green fluorescence were added to the RhB-PEG polymer at a concentration of 1 mg/mL. Scale bars, 5 mm (left) and 1 mm (right). **b**, Optical image of the MEF/CdSe-ZnS/RhB-PEG probe in the brain slice at 22 hours post implantation. Scale bar, 500 µm. **c**, Time-dependent red fluorescence (RhB-PEG 4000, magenta), green fluorescence (CdSe-ZnS nanoparticles, green), and overlay images of the brain slice with the implanted MEF/CdSe-ZnS/RhB-PEG probe. Scale bar, 500 µm.

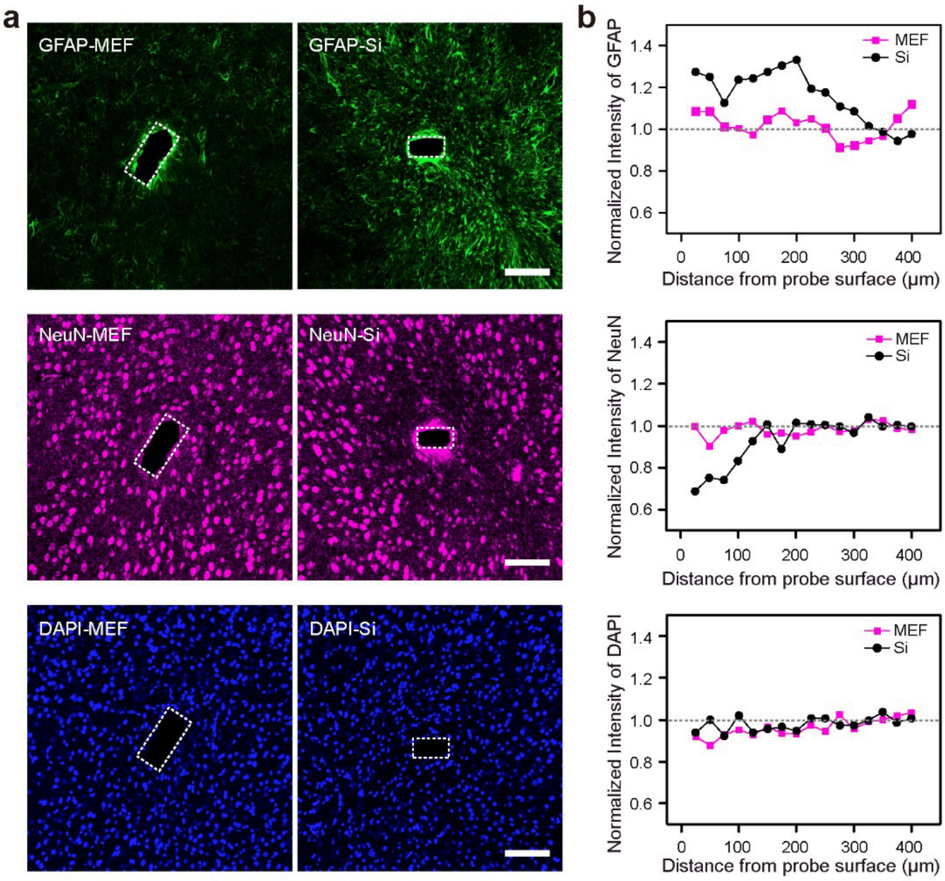

Supplementary Fig. 5. Chronic tissue responses to MEFs at 5 weeks after implantation. a, Immunohistochemical staining images of a brain slice with an implanted self-assembled MEF probe (left) and a silicon probe (right). The 100-μm-thick brain slice was labeled for astrocytes [glial fibrillary acidic protein (GFAP), green], neurons (NeuN, magenta), and nuclei [4', 6-diamidino-2-phenylindole (DAPI), blue]. The edges of the MEFs and the silicon probe in the staining images were highlighted by white dashed lines. Scale bar, 100 μm. **b**, Normalized fluorescence intensity of GFAP, NeuN, and DAPI around the MEFs and the silicon probe.

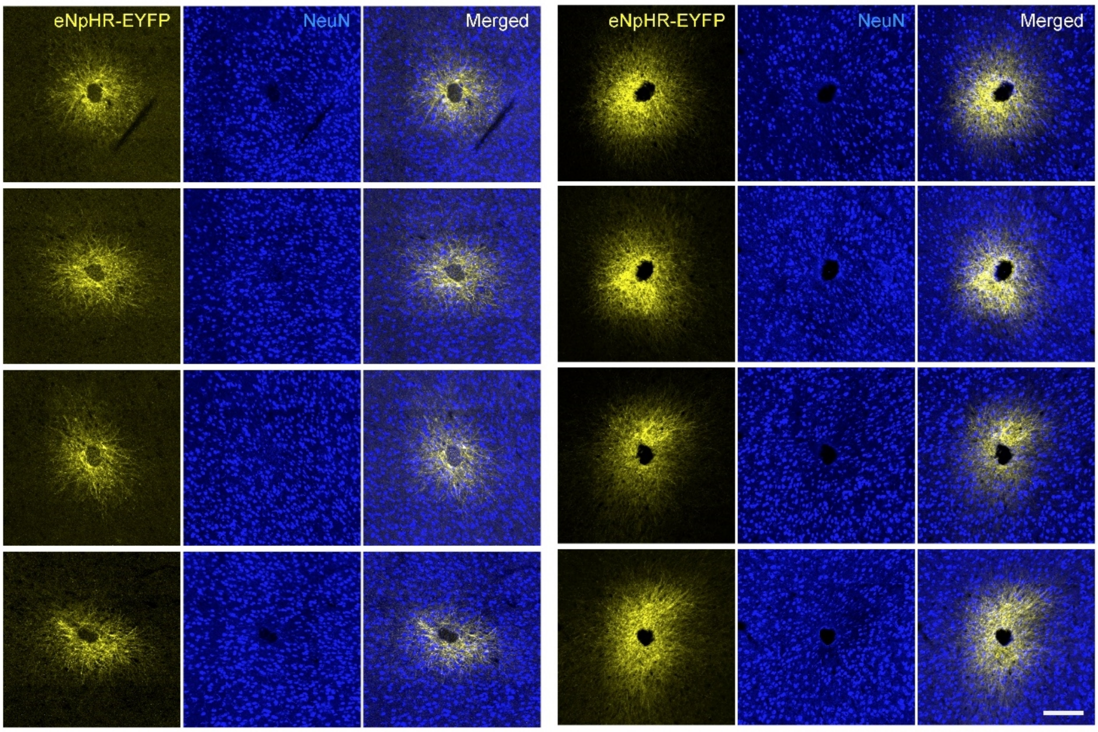

Supplementary Fig. 6. Neuronal transduction of self-assembled probes. Brain slices were characterized at 3 weeks after implantation of AAV9-hSyn::eNpHR3.0-EYFP-delivery probes. NeuN was used to label the nuclei of mature neurons in the brain slices, and $NeuN^+$ cells were identified using ImageJ software. The transduction efficiency of neurons, *i.e.* the ratio between the number of $EYFP^+NeuN^+$ cells and the total number of $NeuN^+$ cells, was 91 ± 11% within 50 μm from the microelectrodes. Scale bar, 200 μm.

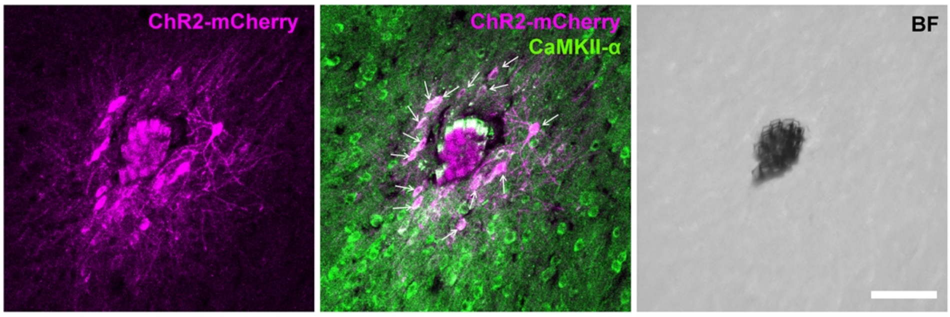

Supplementary Fig. 7. Opsin expression in excitatory neurons at microelectrode-tissue interfaces. ChR2-mCherry expression was analyzed at 3 weeks after an AAV9-CaMKIIα::ChR2-mCherry-delivery probe was implanted in a mouse brain. White arrows highlight ChR2-mCherry^{+}CaMKIIα^{+} cells. Excitatory neurons were stained with rabbit anti-CaMKII (1:200) and goat anti-rabbit Alexa-405 (1:500). Scale bar, 100 μm.

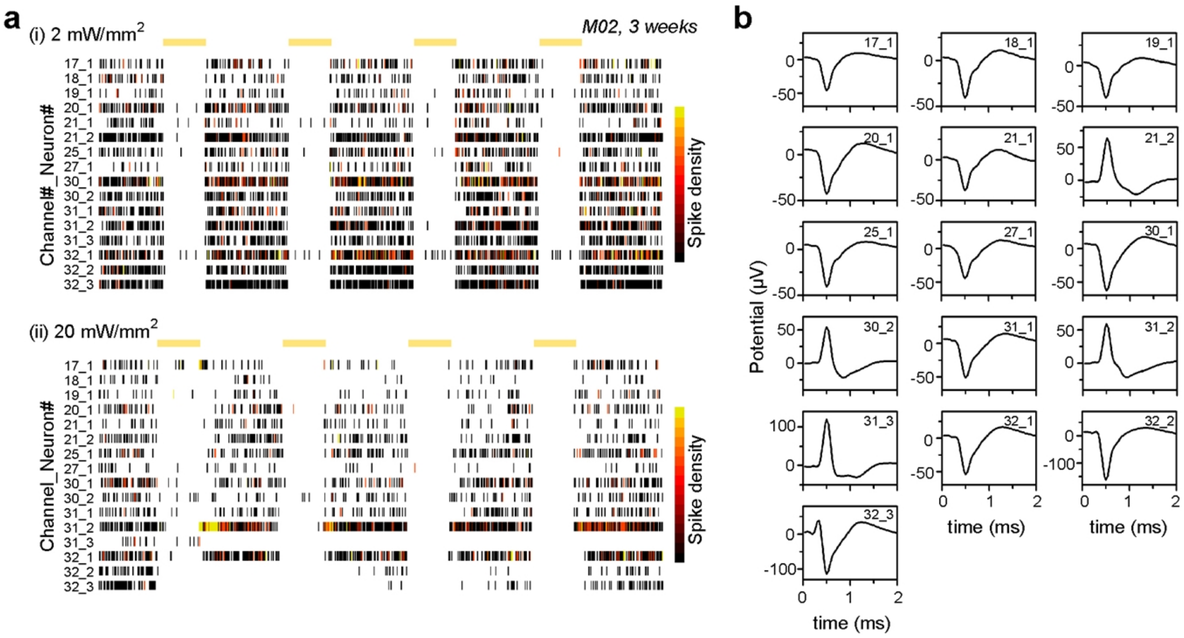

Supplementary Fig. 8. Optogenetic inhibition and electrical recording in mouse M02 at 3 weeks after implantation. a, Spike density of 16 isolated neurons during 4 trials of 10-s yellow light illumination at laser power density of 2 (i) and 20 mW/mm^2 (ii), respectively. A self-assembled AAV9-hSyn::eNpHR3.0-EYFP-delivery probe was implanted in the M2/VO cortex of mouse M02. The light duration of each stimulation was indicated with yellow bars. Color represents the relative number of spikes generated in each time bin, where yellow and black correspond to the maximum and minimum number, respectively. **b**, Averaged spike waveforms of 16 isolated neurons in mouse M02.

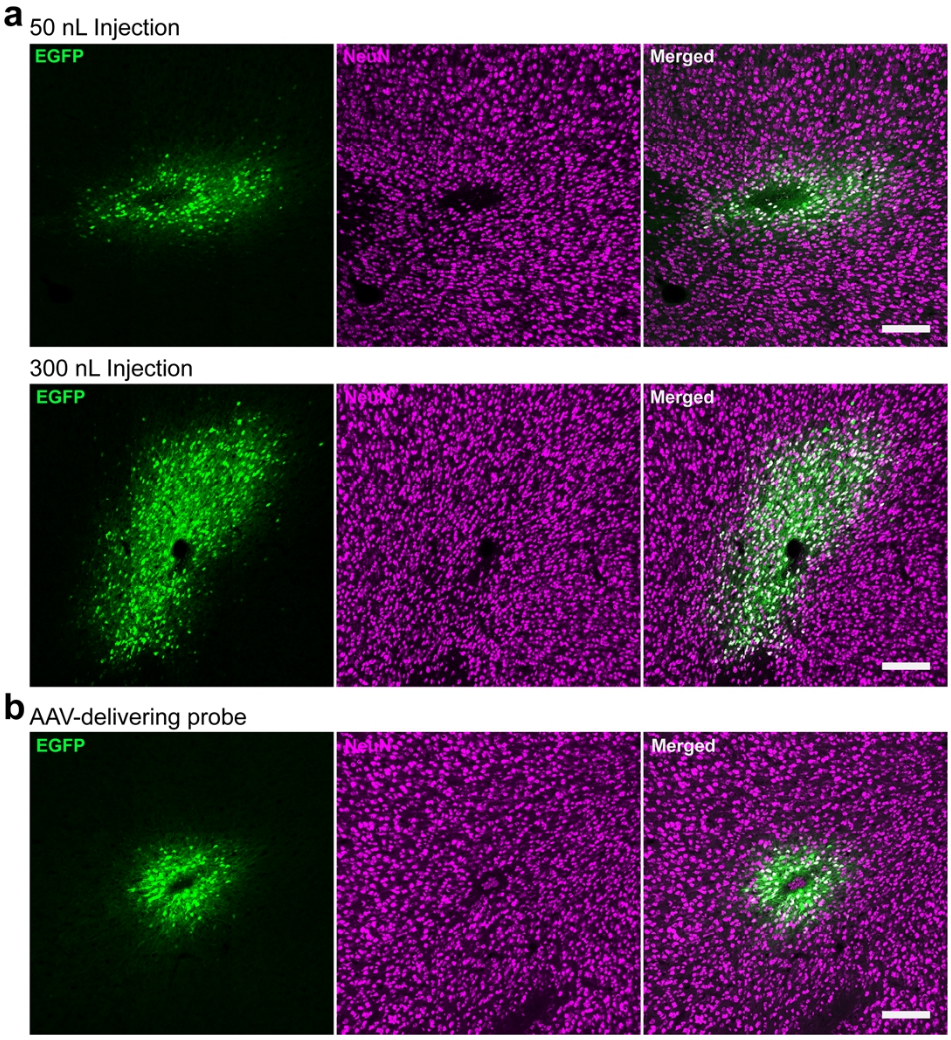

Supplementary Fig. 9. Transduction comparison between self-assembled probes and solution injections. a, EGFP expression of 50-nL and 300-nL AAV9-hSyn::EGFP solution injections at 3 weeks. Scale bars, 200 μm. **b**, EGFP expression of a self-assembled AAV9-hSyn::EGFP-delivery probe at 3 weeks after implantation. Scale bar, 200 μm.

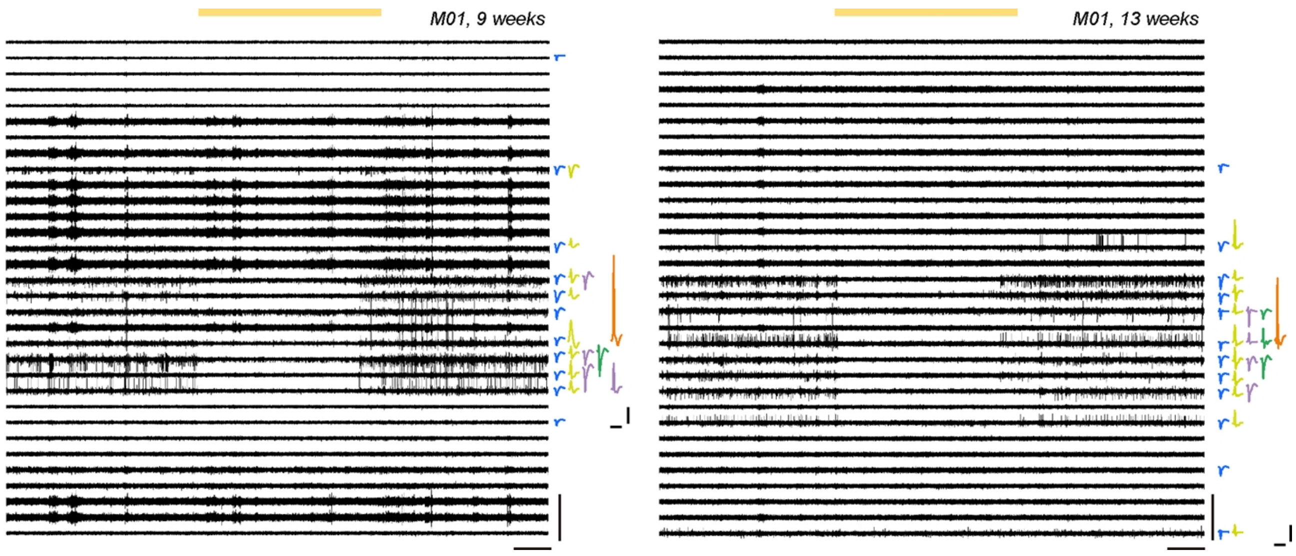

Supplementary Fig. 10. Long-term optogenetic inhibition and electrical recording in mouse M01. AP traces (250 to 5000 Hz) recorded by an implanted AAV9-hSyn::eNpHR3.0-delivery probe at 9 weeks (left, from which 25 putative individual neurons were isolated) and 13 weeks (right, from which 30 putative individual neurons were isolated), respectively, in mouse M01. Scale bar, 500 µV (vertical) and 2 s (horizontal) for AP traces, and 100 µV (vertical) and 2 ms (horizontal) for spikes.

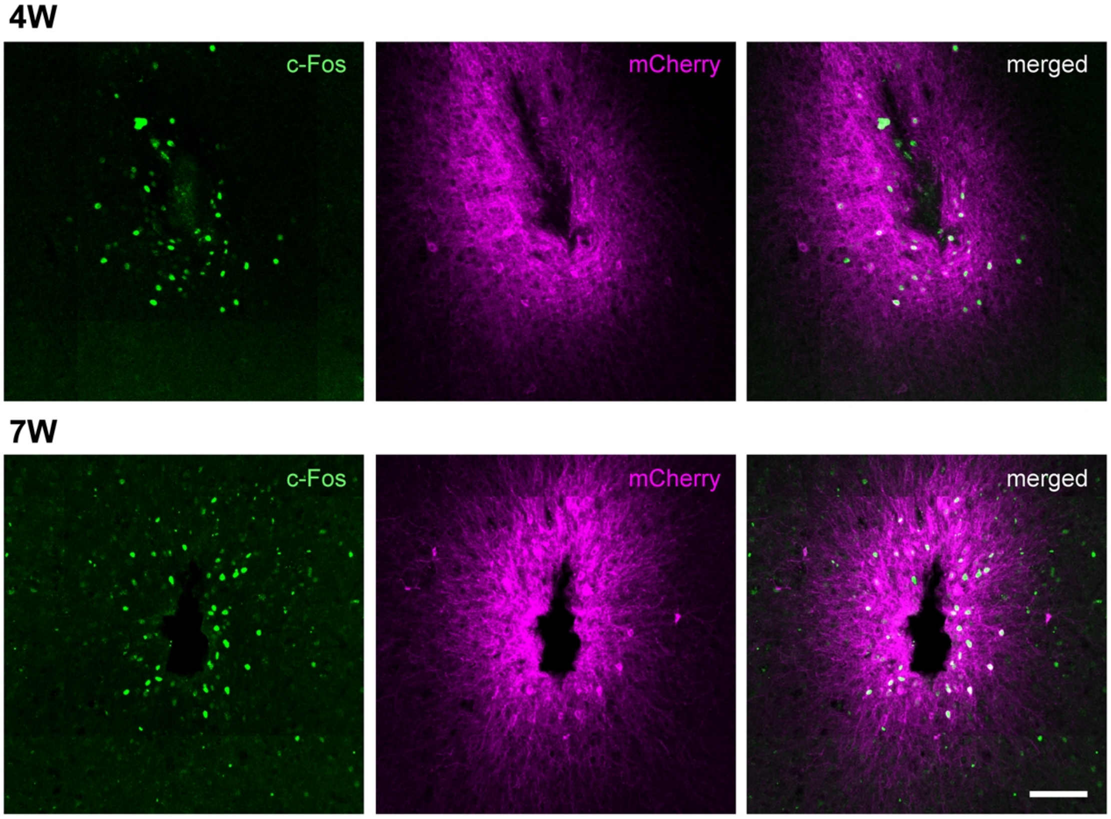

Supplementary Fig. 11. Localized c-Fos activation at microelectrode-tissue interfaces. AAV-delivering optrodes were implanted into the M2/VO regions of wild-type mice. At 4 or 7 weeks post-implantation, 20 min of 10-Hz photostimulation (10-ms pulses, 20-mW/mm^2 power density) was delivered with the AAV-delivering optrodes. Animals were sacrificed 1.5 hour later, and 30-µm-thick brain slices were prepared and stained for c-Fos. Scale bars, 100 µm.

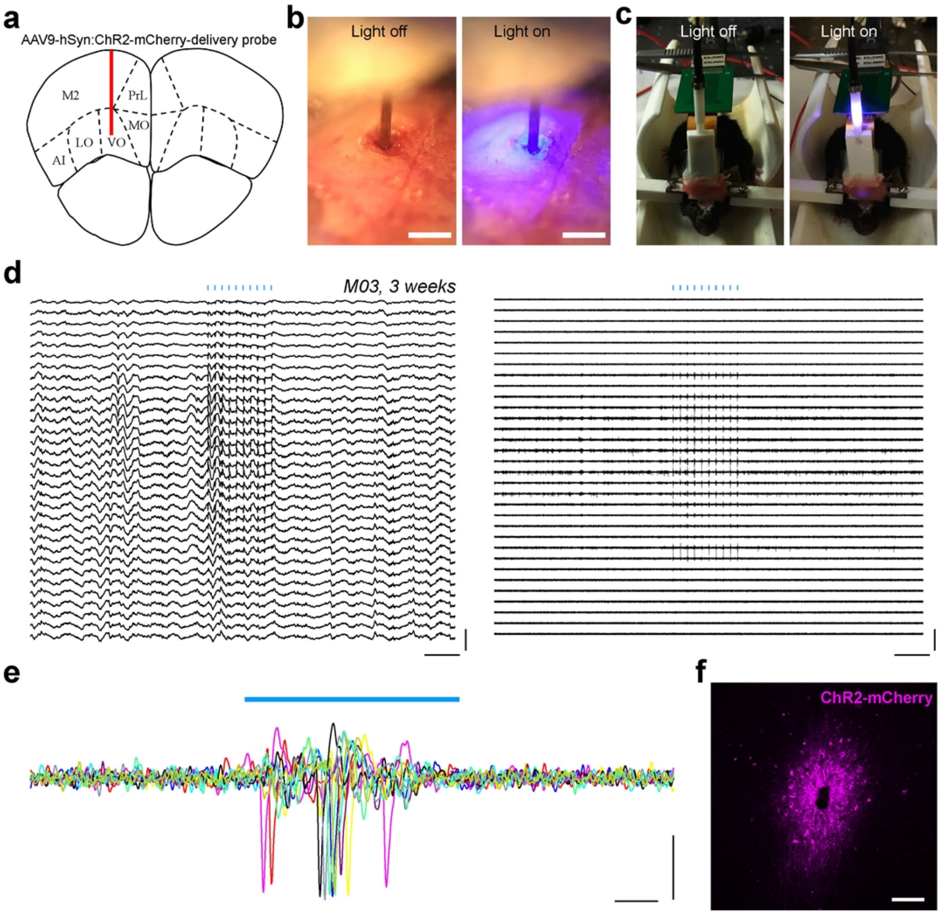

Supplementary Fig. 12. Simultaneous optogenetic activation and electrical recording by implanted AAV9-hSyn::ChR2-mCherry-delivery probes in mouse brains. a, Schematic showing an implanted AAV9-hSyn::ChR2-mCherry-delivery probe in a mouse brain. **b**, Photos of an implanted probe without (left) and with (right) blue light illumination. Scale bar, 1 mm. **c**, Simultaneous optical stimulation and electrical recording with a AAV9-hSyn::ChR2-mCherry-delivery probe implanted in the M2/VO cortex of a mouse brain. **d**, Multi-channel LFP (left, low pass 250 Hz) and AP traces (right, 250 to 5,000 Hz) recording in mouse M03 at 3 weeks after implantation. Blue light pulses were delivered for 1 s and indicated as blue bars. The parameters of the blue light pulses were: 10-ms pulse width, 10 Hz frequency, and 20 mW/mm^2 power density. Scale bar, 1 mV (vertical, left), 500 ms (horizontal, left), 400 µV (vertical, right), and 500 ms (horizontal, right). **e**, Overlay electrical signals from 10 repeated optogenetic stimulation trials of a representative channel (Ch24, M03). AP traces were filtered in the 250~5,000 Hz frequency range from raw recording data in response to 10 Hz, 10-ms blue light pulses. Scale bars, 2 ms (horizontal), 100 µV (vertical). **f**, Opsin expression of an AAV9-hSyn::ChR2(H134R)-mCherry-delivery probe at 8 weeks. Scale bar, 100 µm.

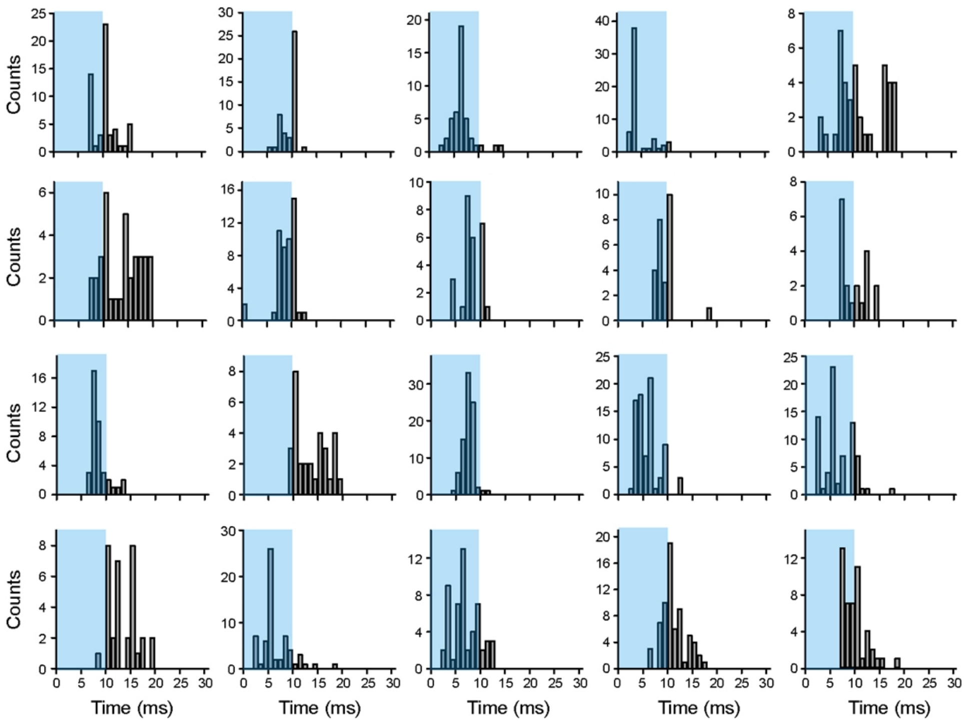

Supplementary Fig. 13. Latency between the light onset and the first spike of ChR2-expressing neurons at 3 weeks post implantation. The parameters of the blue light pulses were: 10-ms pulse width, 10 Hz frequency, and 20 mW/mm^2 power density. Blue boxes indicate the 10-ms laser pulses.

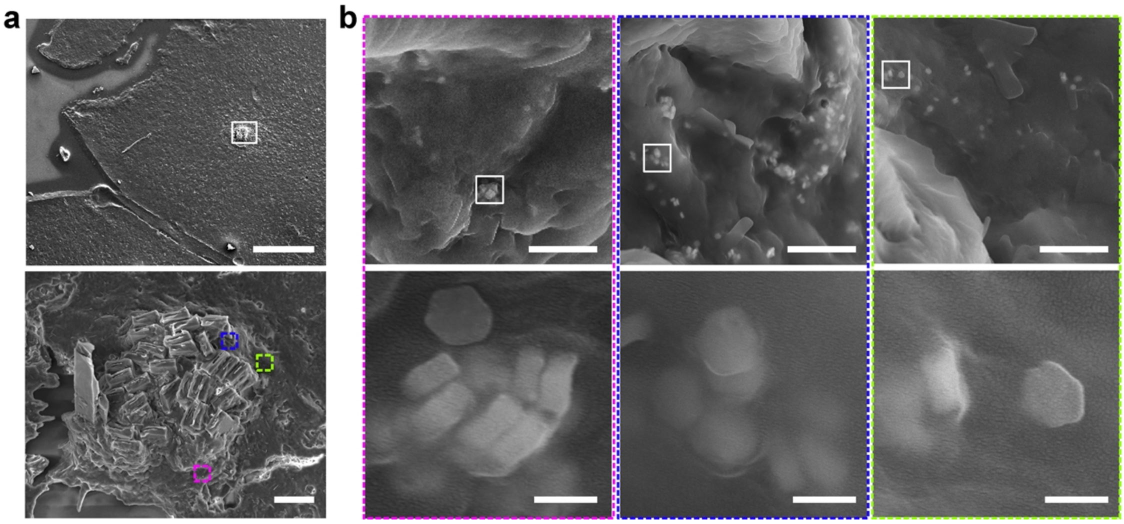

Supplementary Fig. 14. A brain slice with an implanted nanoparticle-delivery probe. **a**, SEM image of a brain slice with an implanted nanoparticle-delivery probe. Bottom is the enlarged view in the white box of the top image. Scale bars, 500 μm (top) and 20 μm (bottom). **b**, Enlarged SEM images in the dashed boxes in **a**. Scale bars, 2 μm (top) and 200 nm (bottom).

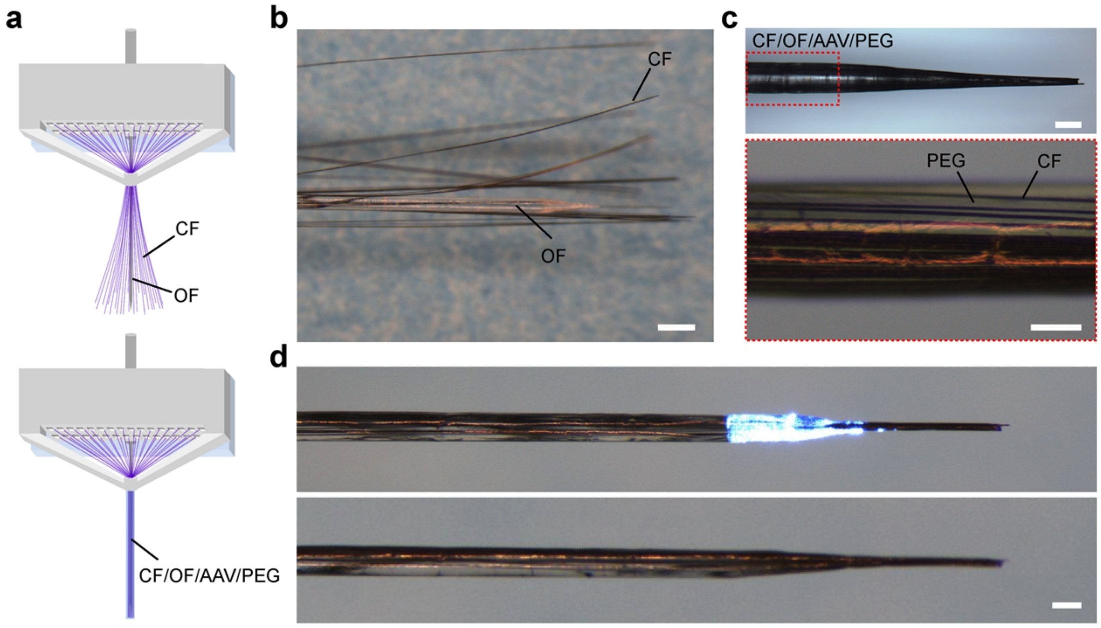

Supplementary Fig. 15. VVD-optrode based on carbon fiber (CF) microelectrodes. a, Schematic showing the construction of a VVD-optrode using flexible CF microelectrodes. **b**, Optical fiber and 32 CF microelectrodes dispersed in water. The CF microelectrodes have a diameter of 7 µm and can be obtained from ANATECH (China). Scale bar, 500 µm. **c**, A self-assembled CF/OF/AAV/PEG probe. Scale bars, 200 µm (top) and 100 µm (bottom). **d**, A self-assembled CF/OF/AAV/PEG probe without (top) and with blue light illumination (bottom), respectively. Scale bar, 200 µm.
