# Supplementary material for: Self-assembled multifunctional neural probes for precise integration of optogenetics and electrophysiology
Source: Nat Commun. 2021 Oct 7;12:5871. doi: 10.1038/s41467-021-26168-0 (PMC8497603; doi:10.1038/s41467-021-26168-0)
Supplement: Supplementary file 2 — Reporting Summary [file 41467_2021_26168_MOESM2_ESM.pdf]

nature research

Corresponding author(s): Ying Fang

Last updated by author(s): Aug 19, 2021

Reporting Summary

Nature Research wishes to improve the reproducibility of the work that we publish. This form provides structure for consistency and transparency in reporting. For further information on Nature Research policies, see our Editorial Policies and the Editorial Policy Checklist.

Statistics

For all statistical analyses, confirm that the following items are present in the figure legend, table legend, main text, or Methods section.

n/a	Confirmed	
☐	☒	The exact sample size (n) for each experimental group/condition, given as a discrete number and unit of measurement
☐	☒	A statement on whether measurements were taken from distinct samples or whether the same sample was measured repeatedly
☐	☒	The statistical test(s) used AND whether they are one- or two-sided<br>*Only common tests should be described solely by name; describe more complex techniques in the Methods section.*
☒	☐	A description of all covariates tested
☒	☐	A description of any assumptions or corrections, such as tests of normality and adjustment for multiple comparisons
☐	☒	A full description of the statistical parameters including central tendency (e.g. means) or other basic estimates (e.g. regression coefficient) AND variation (e.g. standard deviation) or associated estimates of uncertainty (e.g. confidence intervals)
☒	☐	For null hypothesis testing, the test statistic (e.g. F, t, r) with confidence intervals, effect sizes, degrees of freedom and P value noted<br>*Give P values as exact values whenever suitable.*
☒	☐	For Bayesian analysis, information on the choice of priors and Markov chain Monte Carlo settings
☒	☐	For hierarchical and complex designs, identification of the appropriate level for tests and full reporting of outcomes
☒	☐	Estimates of effect sizes (e.g. Cohen's d, Pearson's r), indicating how they were calculated

Our web collection on statistics for biologists contains articles on many of the points above.

Software and code

Policy information about availability of computer code

Data collection	Olympus LEXT OLS4000 version 2.2.3, Carl Zeiss ZEN 2010B SP1 version 6.0.0.485, Central application version 7.0.16.0.
Data analysis	Carl Zeiss ZEN 2.3 SP1 (black) version 14.0.0.201, ImageJ 1.47v, Offline Sorter application version 4.4.0, NeuroExplorer data analysis application version 5.1.1.8, Matlab R2009b.

For manuscripts utilizing custom algorithms or software that are central to the research but not yet described in published literature, software must be made available to editors and reviewers. We strongly encourage code deposition in a community repository (e.g. GitHub). See the Nature Research guidelines for submitting code & software for further information.

Data

Policy information about availability of data

All manuscripts must include a data availability statement. This statement should provide the following information, where applicable:

- Accession codes, unique identifiers, or web links for publicly available datasets
- A list of figures that have associated raw data
- A description of any restrictions on data availability

The electrophysiology data generated in this study have been deposited on Figshare: https://doi.org/10.6084/m9.figshare.c.5055200.v1. Data used to plot Fig. 2b, Fig. 3d-f, Fig. 4b,f, Supplementary Fig. 5b, Supplementary Fig. 6, and Supplementary Fig. 13 in this paper have been provided in the Source Data file. Additional raw data generated in this study are available from the corresponding author upon reasonable request.

Field-specific reporting

Please select the one below that is the best fit for your research. If you are not sure, read the appropriate sections before making your selection.

☒ Life sciences ☐ Behavioural & social sciences ☐ Ecological, evolutionary & environmental sciences

For a reference copy of the document with all sections, see nature.com/documents/nr-reporting-summary-flat.pdf

Life sciences study design

All studies must disclose on these points even when the disclosure is negative.

Sample size	Sample sizes were determined by our previous work or similar work in the literature.
Data exclusions	Mice that failed to survive for 2 weeks following surgery were excluded.
Replication	The number of independently replicated experiments is described in manuscript. All replication attempts were successful.
Randomization	Animals were assigned randomly into experimental groups.
Blinding	Electrophysiology data were not collected in a blind way. The lead investigator of this study was responsible for both animal surgery and data collection, blinding was therefore impractical. For fluorescent and immunochemical analysis, the investigators were blinded to group assignment.

Reporting for specific materials, systems and methods

We require information from authors about some types of materials, experimental systems and methods used in many studies. Here, indicate whether each material, system or method listed is relevant to your study. If you are not sure if a list item applies to your research, read the appropriate section before selecting a response.

Materials & experimental systems

n/a	Involved in the study	
☐	☒	Antibodies
☒	☐	Eukaryotic cell lines
☒	☐	Palaeontology and archaeology
☐	☒	Animals and other organisms
☒	☐	Human research participants
☒	☐	Clinical data
☒	☐	Dual use research of concern

Methods

n/a	Involved in the study	
☒	☐	ChIP-seq
☒	☐	Flow cytometry
☒	☐	MRI-based neuroimaging

Antibodies

Antibodies used	Monoclonal mouse anti-NeuN (Millipore, MAB377, 1:200), goat anti-mouse Alexa-633 (Thermo Scientific, A-21050, 1:1000), goat anti-mouse Alexa-405 (Thermo Scientific, A-31553, 1:1000), polyclonal rabbit anti-GFAP (Sigma, HPA056030, 1:1000), goat anti-rabbit Alexa-488 (Thermo Scientific, A-11008, 1:1000), polyclonal rabbit anti-CAMKIIa (GeneTex, GTX127939, 1:200), polyclonal rabbit anti-c-Fos (SYSY, 226003, 1:1000), goat anti-rabbit Alexa-405 (Thermo Scientific, A-31556, 1:500),DAPI (Beyotime, C1002, 1:1000). All antibodies were used according to the profile of manufacturer.
Validation	Monoclonal mouse anti-NeuN is suitable for FC/ICC/IF/IHC-P/IP/WB (species reactivity: human, salamander, pig, chicken, mouse, ferret, avian, rat), polyclonal rabbit anti-GFAP is suitable for IB/IF/ICC (species reactivity: human, mouse), polyclonal rabbit anti-CAMKIIa is suitable for WB/ICC/IF/IHC-Fr (species reactivity: mouse, rat), polyclonal rabbit anti-c-Fos is suitable for WB/ICC/IHC (species reactivity: human, rat, mouse, monkey, ape, cow, dog, pig).

Animals and other organisms

Policy information about studies involving animals; ARRIVE guidelines recommended for reporting animal research

Laboratory animals	Male C57BL/6 mice (Vital River Laboratory Animal Technology Co. Ltd., Beijing, China) were used. The mice were 7-8 weeks old and weighed 20-30 g at the time of viral injection and probe implantation. Mice were housed and maintained at 22 ℃， 12 h dark/light cycle, and 50% humidity with provided food and water ad.
Wild animals	The study did not involve wild animals.
Field-collected samples	The study did not involve samples collected from the field.

Ethics oversight	All animal procedures were approved and oversighted by the by the Animal Care and Use Committee of the National Center for Nanoscience and Technology, China.

Note that full information on the approval of the study protocol must also be provided in the manuscript.
